# Quantifying the Delays Between Multi-Site Photoplethysmography Pulse and Electrocardiogram R-R Interval Changes Under Slow-Paced Breathing

**DOI:** 10.3389/fphys.2019.01190

**Published:** 2019-09-25

**Authors:** John Allen

**Affiliations:** ^1^Microvascular Diagnostics, Northern Medical Physics and Clinical Engineering Department, Freeman Hospital, Newcastle upon Tyne, United Kingdom; ^2^Faculty of Medical Sciences, Institute of Cellular Medicine, Newcastle University, Newcastle upon Tyne, United Kingdom

**Keywords:** autonomic function, paced breathing, photoplethysmography, pulse arrival time, respiration, R-R interval

## Abstract

**Objective:** Objective assessment of autonomic function is important, including the investigation of slow-paced breathing to induce associated periodic changes in the cardiovascular system – such as blood pressure and heart rate. However, pulse changes across a range of peripheral body sites have seldom been explored with this challenge. The primary aim of this pilot study was to utilize multi-site photoplethysmography (MPPG) technology to quantify the phase delays, i.e., correlation lags, between changes in heart rate and changes in key pulse features with slow-paced breathing (0.1 Hz).

**Methods:** Waveforms were collected simultaneously from the right and left ear lobes, thumbs, and great toes of 18 healthy adult subjects. Cross correlation lags between reference beat-to-beat changes in electrocardiogram (ECG) R-R wave interval and changes in pulse arrival time (foot of pulse; PATf) and also for pulse amplitude (foot-to-peak; AMP) were determined.

**Results:** Relative to R-R changes, the median ear, thumb, and toe PATf correlation lags were 3.4, 2.9, and 2.1 beats, respectively; contrasting to AMP with 5.7, 6.0, and 6.9 beats, respectively. These PATf correlation lags in beats were significantly lower than for the AMP measure. Segmental differences between sites and timing measure variability have also been quantified.

**Conclusion:** This pilot study has indicated bilateral similarity plus segmental differences for relative delays in PPG pulse timing and amplitude measures relative to R-R interval changes with paced breathing. These correlation and variability data are now available for comparison with cardiovascular patient groups to support development of autonomic function assessment techniques.

## Introduction

A pulse is transmitted to the periphery with each heartbeat, with propagation characteristics influenced by aging and/or cardiovascular disease. The pulse can be detected non-invasively using photoplethysmography (PPG), typically utilizing a near-infrared optical transducer to produce a signal associated with red blood cell changes in the tissue micro-vascular bed. PPG signals can be complex, and they have high-frequency (pulse shape; “AC”) and low-frequency (e.g. vasomotion; “DC”) components (Nitzan et al., 1999; Allen and Murray, 2000a; Allen, 2007). PPG characteristics are body site specific with differences reported in timing (pulse arrival time, PAT) (Allen and Murray, 2000b,c) and amplitude (Nilsson et al., 2007; Nilsson, 2013), as well as in their variability (Nitzan et al., 1998; Chen et al., 2015; Bentham et al., 2018).

Innovative multi-site PPG technology (MPPG) has been developed to study the circulation using peripheral pulse assessments from ear, thumb (finger), and great toe sites (Allen and Murray, 2000a). MPPG has shown value in the study of changes in pulse with aging (Allen and Murray, 2002), peripheral arterial disease (Allen et al., 2008; Bentham et al., 2018), connective tissue disease (McKay et al., 2014), endothelial function (Selvaraj et al., 2009), and arterial stiffness (Sharkey et al., 2018). Autonomic function assessment is also of great interest, including using PPG to track respiration rate (Johansson and Öberg, 1999a,b; Ovadia-Blechman et al., 2017). In normal healthy subjects breathing slowly and regularly, e.g., at 0.1 Hz, the heart rate and blood pressure can synchronize to this driving frequency (Estañol et al., 2008; Kobayashi, 2009; Chen et al., 2015; Cernes and Zimlichman, 2017; Ovadia-Blechman et al., 2017). PPG characteristics can also be modulated in this way (Allen and Murray, 2000b,c) but noting that only limited data have been published so far, i.e., relationship between finger pulse transit time and cardiac electrocardiogram (ECG) R-R interval changes (Drinnan et al., 2001). As far as the author is aware, there have been no comparative published studies quantifying the phase relationships between simultaneous head-to-foot PPG pulse timing and amplitude changes and R-R interval changes, although such data could ultimately yield valuable information for cardiovascular system assessment. The primary aim of this pilot study was to quantify the correlation lags between MPPG pulse timing/amplitude changes and R-R interval changes under a standardized slow-paced breathing challenge.

## Materials and Methods

### Physiological Measurements

The MPPG system has been described previously for cardiovascular assessment (Allen and Murray, 2000a; Allen, 2007). Briefly, PPG pulses were collected simultaneously from the right and left ear lobes and pads of thumbs/great toes using six matched PPG amplifiers (bandwidth 0.005–30.0 Hz). Reflection mode probes (Artema, Denmark: ear type 75331-9 with clip, thumb, and toe probes with black Velcro cuff 75333-5) were utilized. A single-lead diagnostic bandwidth ECG provided a cardiac timing reference.

Measurements were collected in a warm and comfortable vascular measurement facility (ambient temperature ~24°C) with subjects positioned supine. Following acclimatization, subjects were asked to perform a slow-paced breathing exercise (one breath every 10 s; inspiration:expiration 50:50) as visually directed by a triangular rolling waveform, with waveforms captured to computer (100 s, sampling rate 500 Hz). Participants had no known cardiovascular disease/arrhythmia. Waveforms were collected in 1999 from healthy staff/student volunteers with informed consent obtained in compliance with best practice at that time. This study is a secondary analysis of this previously collected anonymized data; their further consent was not essential for this study.

### Waveform Analysis

**Figure 1:**
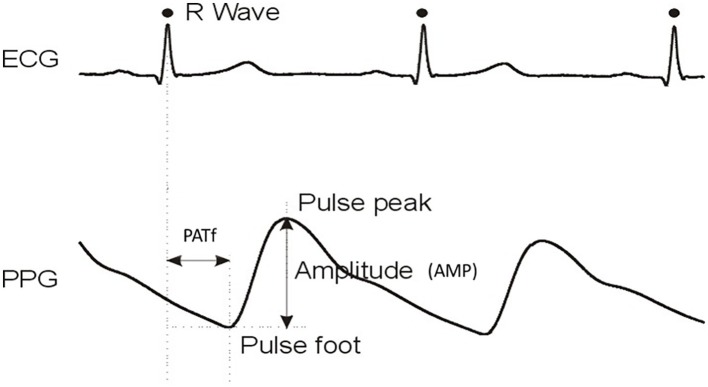
Example ECG and single-site PPG waveforms with key analysis landmarks PATf and AMP identified. PATf and AMP values on a beat-to-beat basis are also determined for the other five body sites of the six-channel MPPG pulse measurement system.

Waveforms were analyzed off-line (MATLAB v2012a, MathWorks) using digital signal processing stages of low/high pass filtering and landmark extraction, then quantification of beat-to-beat ECG R-R interval variation, pulse arrival time (to pulse foot; PATf), and pulse foot-to-peak amplitude (AMP; Figure 1). Analysis was semi-automated and included a manual quality checking stage to allow editing of pulses with poor automatically recognized landmarks. The checked beat-to-beat R-R intervals, PATf, and AMP data were resampled using simple interpolation (resolution 1/100^th^ of a heartbeat). Two sets of analysis were performed: cross correlation and the quantification of PATf and R-R interval variability.

#### Cross Correlation Analysis

MATLAB-based cross correlation analysis determined the delays, i.e., correlation lags between PATf changes and AMP changes for each of the six body measurement sites, relative to R-R interval variation, during slow-paced breathing. The first clear correlation lag peak per body site was semi-automatically determined from cross correlation data for PATf and also for AMP using plots in up to a 12-beat window, measured in beats. Clearly irregular cross correlation plots were excluded.

#### PATf and R-R Interval Timing Variability Analysis

Standard deviations (SDs) for site PATf changes were determined (SD-PATf: Ears; Thumbs; Toes). In addition, normalized variability was also calculated for each site using SD-PATf divided by the cardiac timing reference SD of R-R interval variability (SD-RR) over the same measurement period.

### Statistical Analysis

Minitab (V17) statistical software was utilized. Data were summarized using non-parametric, i.e., median [interquartile range, (IQR)] measures. Bilateral similarity for cross correlation data, timing variability between each of the three-pair body sites, and the segmental differences between sites (Ears-Thumbs; Ears-Toes; Thumbs-Toes) were tested using Wilcoxon’s signed rank test. Unpaired data comparisons utilized the Mann-Whitney *U* test. Differences between segmental levels for PATf and AMP lags and for timing variability measures were assessed using Kruskal-Wallis test. Standard linear regression tested for significant associations of age with correlation/timing measures. *p* < 0.05 was considered statistically significant.

## Results

Eighteen healthy adult volunteers [12 male, median age 35 (30–47) years] participated. All could be included for the PATf correlation and normalized variability analyses, but two were excluded from the AMP analysis as their correlation plots could not be interpreted with confidence. Median (IQR) peak cross correlation levels between R-R intervals and PATf for Ears, Thumbs, and Toes were 0.55 (0.39–0.70), 0.62 (0.45–0.76), and 0.71 (0.60–0.75), respectively, and those levels between R-R intervals and AMP for Ears, Thumbs, and Toes were 0.61 (0.41–0.73), 0.34 (0.25–0.57), and 0.39 (0.22–0.51), respectively.

**Figure 2:**
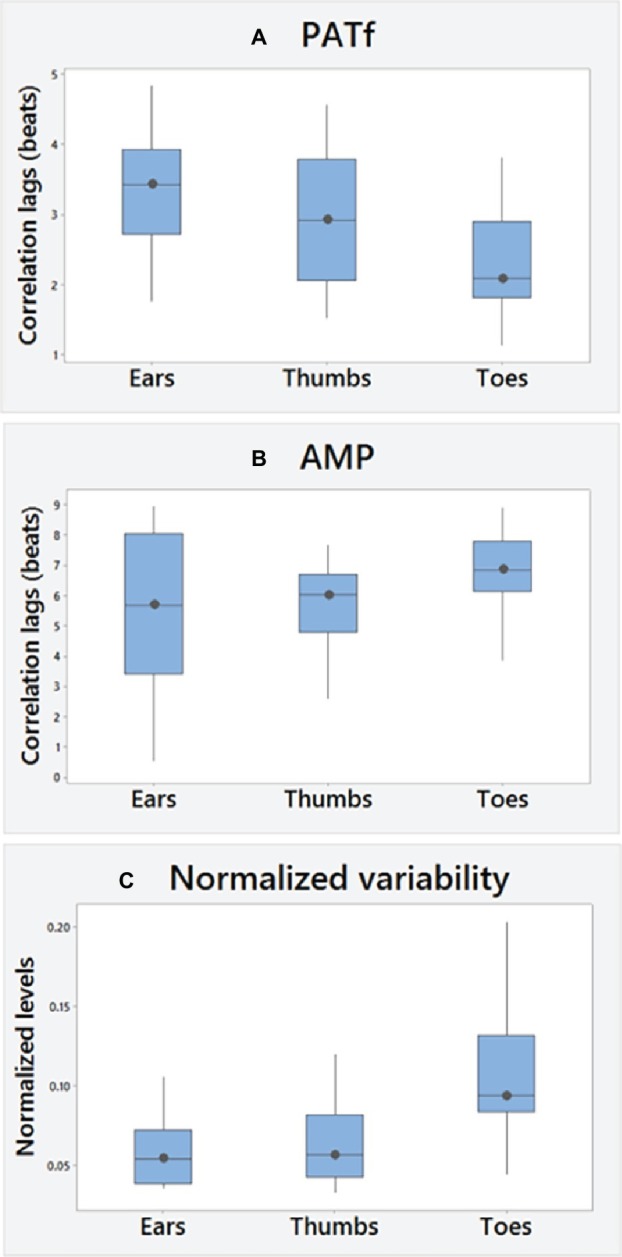
Overall **(A)** PATf and **(B)** AMP correlation lags and **(C)** PATf timing variability normalized to cardiac changes (SD of PATf at each site normalized using the SD of R-R intervals). A positive cross correlation value, i.e., lag means changes in PPG measures lag behind changes in the ECG R-R interval measure during paced breathing.

No significant differences were found between right and left PATf correlation lags for any segmental site (Table 1), demonstrating bilateral similarity overall. There was also a tendency to bilateral similarity for the AMP lags for the Ears and Toes, although the right thumb was delayed relative to the left by 0.72 heartbeats (−0.17–1.64; *p* < 0.05). PATf variability (SD-PATf) also showed bilateral similarity. Overall ranges for correlation lags and PPG variability (e.g., SD-PATf) normalized to R-R interval variability (SD-RR) are summarized in Figures 2A–C, using the mean of right and left body sides.

**Table 1:** Summary of PATf and AMP cross correlation phase lag data (in heartbeats) and also normalized variability data for the 0.1 Hz paced breathing exercise.

		Right body side	Left body side	*p*
**Correlation phase lags**				
PATf (beats)	Ears	3.57 (2.90–3.82)	3.43 (2.82–4.29)	ns
	Thumbs	2.91 (2.00–3.85)	3.05 (2.07–3.75)	ns
	Toes	2.26 (1.74–2.89)	2.16 (1.81–2.80)	ns
AMP (beats)	Ears	6.85 (3.47–8.22)	5.69 (3.11–8.12)	ns
	Thumbs	6.53 (5.28–7.39)	5.49 (1.78–6.47)	p < 0.05
	Toes	7.09 (6.07–7.44)	6.69 (5.98–8.09)	ns
**SD-PATf variability and normalized to SD-RR**				
Variability (ms)	Ears	4.3 (3.3–5.4)	4.9 (3.3–5.5)	ns
	Thumbs	4.5 (3.9–6.8)	4.4 (3.8–6.4)	ns
	Toes	7.4 (5.9–10.3)	7.4 (6.4–9.2)	ns
Normalized	Ears	0.054 (0.039–0.077)	0.057 (0.037–0.073)	ns
	Thumbs	0.062 (0.042–0.073)	0.056 (0.042–0.077)	ns
	Toes	0.099 (0.076–0.132)	0.094 (0.085–0.142)	ns

*The median phase lag values shown are all positive, i.e., the PPG measure lags the ECG R-R interval measure. Note: ns denotes non-significant*.

There were significant differences overall in PATf correlation lags between segmental levels (*p* < 0.01). These were the lowest for the Toes and with significant differences found between Thumbs and Toes [Thumbs-Toes; +0.74 (−0.32–1.29) beats, *p* < 0.05] and between Ears and Toes [Ears-Toes; +0.97 (0.53–1.39) beats, *p* < 0.005]. There were, however, no significant differences for AMP between the segmental levels. AMP correlation lags were significantly greater than for PATf at all sites (Ears, *p* = 0.01; Thumbs and Toes, both *p* < 0.001).

Overall, there were significant differences between segmental levels for SD-PATf (*p* < 0.001). Normalized variability was the largest at the Toes, and significant segmental differences were found between Ears and Toes [Toes-Ears; 0.046 (0.018–0.069), *p* < 0.001] and between Thumbs and Toes [Toes-Thumbs; 0.047 (0.012–0.059) beats, *p* < 0.001] but non-significant for the comparison of Ears-Thumbs. RR interval variability (SD-RR) was 79.1 (54.6–106.5) ms, which significantly decreased with age (*r* = −0.62; *p* < 0.01), but only the Toes normalized variability changed significantly, i.e., increasing with age (*r* = +0.54; *p* < 0.05). Age-related associations were not found for correlation lag at any site, although PATf peak correlation levels decreased at proximal sites with age [ears (*p* < 0.01); Thumbs (*p* < 0.05)]. No significant differences were found between male and female subjects.

## Discussion

Cross correlation and variability data have been obtained for MPPG head-to-foot pulse measurements plus ECG during a slow, i.e., 0.1 Hz paced breathing exercise. Previous published MPPG work has shown the tendency to bilateral similarity in healthy subjects across the age ranges in the case of “static” pulse arrival (transit) time and shape. This study has extended the knowledge on PPG by quantifying the relative changes and variability between head-to-foot peripheral sites under this breathing challenge. In healthy subjects, the cross correlation derived delays have the tendency to bilateral similarity, specific segmental level differences, and with differences between the pulse timing and amplitude characteristics.

The key study by Drinnan et al. (2001) reported a mean (SD) delay between pulse timing changes and R-R interval changes of 3.17 (0.76) beats for the single-finger site studied. Corresponding results from this study are consistent but by using MPPG the PATf data add value to show that correlation lags appear to be the shortest for the toes (greatest for the ears). In addition, it has been noted that overall pulse amplitude correlation lags are significantly higher than for PATf. The reasons for the segmental site differences observed are currently unclear but certainly warrant further assessment in both healthy subjects and cardiovascular patients. It may be that the different correlation lags across body sites may be due to the different origins of the PPG variability: autonomic nervous system (sympathetic in toes and fingers and both sympathetic and parasympathetic in ears) and two mechanical effects, which might differently affect lower and upper body sites (Shalom et al., 2013). The degree of variability in pulse under slow-paced breathing has also demonstrated a tendency to bilateral similarity and segmental differences, e.g., with toe site having the largest overall normalized variability. It is possible that this could link to regional differences in sympathetic tone and/or blood vessel compliance. It would be very interesting next to apply the techniques to the study of whole body blood pressure control and cardiovascular coupling. The reducing peak correlation levels with age for proximal site PATf relative to R-R should also be further investigated.

Other vascular optical technologies have been utilized for studying head-to-foot low frequency oscillations under paced breathing, including multi-channel near-infrared spectroscopy (Li et al., 2018). They also found a tendency to bilateral similarity/segmental differences with delays in signals of up to several seconds. The optical technique though studied oxyhemoglobin concentration fluctuations rather than beat-to-beat perfusion with MPPG. There is scope though for future sensor development allowing the parallel assessment of spectroscopy and PPG to take forward in different patient groups. Peak cross correlation level differences between sites and between pulse measures have also been shown – information which could help inform sensor design for respiration rate/blood pressure tracking with PPG based wearable sensors. There are many exciting developments in PPG technology, including MPPG variants, wearable sensors, and non-contact imaging photoplethysmography (iPPG; Sun and Thakor, 2016), which could be used to explore regional signal variations across the body with paced breathing challenges.

### Limitations of Study

Limited demographic data were collected in this pilot – a larger future study should include full demographics to allow appropriate multivariate analyses to be performed; more data from females would help give confidence so that the test is not underpowered for assessing gender differences; cross correlation is a linear function and can be limited in locating phase lags between two time series interacting in a closed loop, and thus, the MPPG signal analysis could benefit from using non-linear techniques (Faes et al., 2013). Future works should also assess measurements from a larger group of normals across the age ranges and explore these with assessments including arterial stiffness and vascular compliance. Furthermore, other novel signal processing approaches could be explored, including: non-linear techniques based on corrected conditional entropy, identifying where MPPG measures are distributed in frequency (e.g., Mayer waves, respiration-induced oscillations), and alternative methods to estimate the gains between signal and variability measures (Maestri et al., 1998; Faes et al., 2013). Data sets could also be collected to contrast a range of breathing frequencies to explore variability magnitude/phase effects between PPG and ECG. Ultimately, we will need to compare normative data sets with cardiovascular patient groups, including patients with diabetes and/or dementia where autonomic and vascular function can be impaired.

## Conclusion

Innovative multi-site photoplethysmography technology has been utilized in this pilot study to characterize the delays for changes in head-to-foot PPG pulse timing/amplitude characteristics relative to ECG R-R interval under slow-paced breathing. The study has confirmed a tendency to bilateral similarity plus segmental differences. Cross correlation and variability data are now available for comparison with cardiovascular patient groups to further support the development of novel autonomic function assessment techniques.

## Ethics Statement

Eighteen healthy staff/students linked to Newcastle University’s Cardiovascular Physics and Engineering Research group were enrolled into the pilot study as volunteers. The MPPG pulse data were originally collected using the non-invasive technology in 2000/1 as part of JA’s PhD research project on photoplethysmography in accordance to a general exploratory protocol and measurement risk assessment. The exclusion criteria were taking cardiovascular medication and/or known cardiovascular disease. All subjects gave informed consent. In the Autumn of 2018, ethical permission was obtained from Newcastle University to reanalyze these anonymized pulse data sets (reference: 7272/2018: “A simple exploratory assessment of the correlations between multi-site PPG pulse characteristics and heart rate during paced breathing”).

## Author Contributions

The author confirms being the sole contributor of this work and has approved it for publication.

### Conflict of Interest Statement

Between 2014 and 2018, Dr. John Allen was the Chief Investigator on an NIHR i4i funded grant (II-C1–0412-20,003) to develop a miniaturized version of multi-site PPG technology – specifically for peripheral arterial disease (PAD) detection in a primary care setting. He is a co-author on two published patents in relation to the GP/primary care MPPG device, i.e., a pulse algorithm for PAD detection and also a novel pulse sensor housing and attachment clip.
